# Migrants: Left behind in health?

**DOI:** 10.1016/j.lanwpc.2021.100119

**Published:** 2021-02-19

In 2019, the UN estimated that more than 272 million international migrants contributed to global economies. The International Organization for Migration has also estimated that 40 million of those migrants live in the Asia-Pacific region. As the largest WHO region, the Western Pacific has seen several COVID-19 clusters in these migrant populations, including in Malaysia and Singapore. However, the outbreaks in this susceptible population did not indicate a novel problem of poor health access for migrants, but instead highlight the importance of accessible public health for all.

Migrant workers face unique challenges such as financial, communication, and legislative barriers to accessing health care. Additionally, their health status is subject to the interaction of a complex set of factors, such as pre-departure health issues, local disease patterns, cultural practices, and socioeconomic status. In 2018, The UCL–*Lancet* Commission on Migration and Health responded to this issue with a framework for addressing the inequity. The Commission highlighted the barriers and facilitators to providing health care, as well as the health impact on migrants. It also described a research agenda to further the field. The Commission has now evolved to become The Lancet Migration global collaboration, which aims to advance the field through research, knowledge dissemination, and advocacy. Ultimately, this collaboration is a strong call for governments and international agencies to provide ethical and feasible migrant health policies to ensure everyone receives quality health care.

WHO defines universal health coverage (UHC) as a guarantee that everyone can access quality health care without enduring financial hardship. Although this goal aims to protect everyone, most countries' commitment to UHC paradoxically leaves out some of the most vulnerable populations, such as migrants. Some governments, such as the US and Australian Governments, have also succumbed to anti-immigration policies and actively disincentivise migrants from seeking health care. Within the Western Pacific region, the presence and coverage of public health-related laws are associated with superior health outcomes such as reduced maternal and neonatal mortality, and fewer HIV infections. Many of these countries, including Cambodia, China, and Vietnam, have also worked on financing and health system reforms to pave the way for UHC. However, although the legislature and finance reforms move the region towards health equity, the inclusion of migrants in the transformation is often unclear.

The ambiguity regarding the inclusion of migrants is not just limited to health policies but also occurs in research. A small amount of research targets migrant health issues and investigates the variations in health-care access. For example, migrants in Australia had a lower understanding of preconception health, and cultural barriers resulted in increased discomfort in seeking information. However, for the most part, migrants are often an afterthought in research: a throwaway line in the discussion indicating that the work does not include them or reasoning for why they could not be included. This challenge might stem from the scarcity of services directed at migrants and exposes flaws in study designs that steer towards non-immigrant populations. Just as research in multicultural societies has begun to adopt a cultural and linguistically diverse approach to participant inclusion, so must migrants be included to truly reflect society's makeup.

The February issue of *The Lancet Regional Health – Western Pacific* includes some Articles addressing health-care inequity. Hyejin Lee and colleagues, and the accompanying Comment from Deepa Dongarwar and Hamisu Salihu, discuss the persistence of socioeconomic disparities in COVID-19 related outcomes, despite the introduction of UHC for all South Koreans in 2020. The research findings also suggested that pre-existing health issues significantly contributed to the inequality, which emphasises that UHC is not an overnight cure for decades of disparity in health-care access. The current issue also includes a Viewpoint from Wanfen Yip and colleagues, who report the strategies used to build a resilient health-care system during the COVID-19 pandemic. Singapore had uncontrolled outbreaks of the virus in migrant worker dormitories. This Article describes the strategy involved in the country's quick response to provide migrant workers with the necessary care and precautions to control the clusters.

These studies are a step in the right direction, but there is still much more to be done. As part of the 2030 Agenda for Sustainable Development, the UN has pledged to ensure that no one is left behind. To work towards this goal, we call on researchers to commit to including migrants in health research, and policy makers to prioritise inclusion of this susceptible group. Thinking about addressing migrant health only when the cracks in our health-care systems are exposed is not enough. Ensuring migrants have equitable access to health care is not only in our self-interest but more importantly, in the interest of humanity.

*The Lancet Regional Health – Western Pacific*

